# Factors associated with testing for HIV in people aged ≥50 years: a qualitative study

**DOI:** 10.1186/s12889-018-6118-x

**Published:** 2018-10-26

**Authors:** Elaney Youssef, Juliet Wright, Valerie Delpech, Kevin Davies, Alison Brown, Vanessa Cooper, Memory Sachikonye, Richard de Visser

**Affiliations:** 1 Brighton and Sussex Medical School, Brighton, UK; 2 Public Health England, London, UK; 3 University College London, London, UK; 4 UK Community Advisory Board (UK-CAB), London, UK; 5 University of Sussex, Brighton, UK

**Keywords:** HIV, Ageing, Older people, Testing, Health care

## Abstract

**Background:**

Despite a decline in the number of new HIV infections in the UK overall, the number and proportion of new HIV diagnoses in people aged ≥50 years continues to increase. People aged ≥50 years are disproportionately affected by late diagnosis, which is associated with poorer health outcomes, increased treatment complexity and increased healthcare costs. Late HIV diagnosis also has significant public health implications in terms of onward HIV transmission. It is not fully understood what factors affect the decision of an older person to test for HIV. The aim of this study was to identify factors associated with testing for HIV in people aged ≥50 years who tested late for HIV.

**Methods:**

We interviewed 20 people aged ≥50 years diagnosed late with HIV to identify factors associated with HIV testing. Interviews were audio recorded, transcribed verbatim and thematically analysed.

**Results:**

Seven themes associated with HIV testing in people aged ≥50 years were identified: experience of early HIV/AIDS campaigns, HIV knowledge, presence of symptoms and symptom attribution, risk and risk perception, generational approaches to health and sexual health, stigma, and type of testing and testing venue.

**Conclusion:**

Some factors associated with testing identified in this study were unique to older individuals. People aged ≥50 years often do not perceive themselves to be at risk of HIV. Further, stigma and a lack of knowledge of how to access HIV testing suggest a need for health promotion and suggest current sexual health services may need to adapt to better meet their needs.

## Background

To halt the AIDS epidemic, the Joint United Nations Programme on HIV/AIDS (UNAIDS) set a target that by 2020, 90% of people with HIV will be aware of their infection, 90% of those on antiretroviral therapy, and 90% of those virally suppressed [1]. In the UK the last two targets have already been exceeded. However public health efforts need to continue to reduce the undiagnosed population [2].

Recently, there has been an observed decline in new HIV diagnoses in the UK mainly among men who have sex with men (MSM) [3]. However the proportion of new HIV diagnoses among people aged ≥50 years has continued to increase [2, 4]. This is a significant public health concern as this group are disproportionately affected by late diagnosis [5]. Individuals diagnosed late with HIV are likely to have been living with undiagnosed HIV infection for several years [6], and pose a significant risk for onward transmission [7]. Further, late diagnosis in this group is associated with significantly poorer health outcomes [8, 9], increased treatment complexity, and increased healthcare costs [10]. By 2028, more than 50% of people living with HIV in the UK will be aged ≥50 years [11]. Furthermore, around half of all new infections among people aged ≥50 years were acquired at age 50+ [9], demonstrating the need for increased efforts to increase HIV testing within this population.

Despite various testing initiatives - including routine screening and home testing or sampling – new diagnoses among people aged ≥50 years diagnosed late with HIV continue to rise. Although sexual activity continues into older age [12–15], older adults often perceive HIV to be a condition which affects younger people. As a consequence, older individuals often do not perceive themselves to be at risk of HIV, resulting in a lack of motivation to seek testing [16–20].

The perception among healthcare providers (HCP) that older people are asexual and therefore not at risk of HIV may act as a barrier to test offer in this group [21–24]. Feeling that older adults are not at risk of HIV may also explain the misattribution of symptoms so that symptoms in an older patient are often attributed to something ‘age-related’ rather than HIV [23, 25]. Further, perceived asexuality in older age may result in HCPs feeling uncomfortable discussing sexual health with older patients [23, 24], hereby creating an additional barrier to HIV test offer in this group. However, despite increased healthcare interactions in older age, they are less likely to be offered HIV testing by a HCP [26].

Although there is some evidence that there are unique factors associated with undergoing HIV testing in older age, it is not yet fully understood why older individuals are less likely than younger individuals to undergo timely HIV testing. The aim of the current study was to identify factors associated with testing for HIV in people aged ≥50 years who tested late for HIV.

## Methods

### Procedure and sample

Recruitment of participants was conducted at 6 HIV clinics across South East England. Purposive sampling ensured inclusion of participants in areas of varying local HIV prevalence, HIV clinic size and patient demographics. Participants were eligible if they were aged ≥50 years at HIV diagnosis, if their diagnosis was late (diagnosed with a CD4 count of < 350 cells/μL or presenting with an AIDS defining event), and if diagnosed within the previous 1–36 months. The upper limit was set strategically to allow for adequate numbers of eligible participants, while not being too long after a diagnosis for adequate recall. In order to maintain confidentiality, eligible participants were identified and approached by a member of their local clinical team. If interested in the study and verbal consent was given, contact details were given to the researcher via telephone, where they were stored in a locked filing cabinet. The lead researcher (EY) contacted people who were interested to arrange a study visit in a private room at the participating HIV clinic, at which written informed consent was obtained. Participants completed a demographic questionnaire which included patient age (in years), and self-identified gender, sexual orientation and ethnicity. Basic clinical variables were also collected, including the presence of AIDS defining illnesses at the time of HIV diagnosis. During the study visit a semi-structured interview was also conducted by EY, lasting approximately 45 min. The initial interview topic guide was based on results of a systematic review which identified patient and clinician-related barriers and facilitators to HIV testing in people aged ≥50 years [27]. However, novel factors associated with HIV testing were explored within the semi-structured approach, and incorporated into subsequent versions of the topic guide. Novel factors included: the impact of early HIV/AIDS campaigns, a theme not found in any previous literature; how ageing affected symptom attribution; and how people perceived their risk of HIV as they aged. Twenty participants were recruited into the study. The sample was diverse: mean age 60 years (range 52–80); 80% (16) identifying as white, 20% (4) identifying as black African/Caribbean; 70% (14) male, 30% (6) female; 60% (12) heterosexual, 30% (6) gay, 10% (2) bisexual; and mean time since HIV diagnosis 17 months (range 4–31).

### Data analysis

Audio recordings of interviews were transcribed verbatim and anonymised (pseudonyms have been used). Transcripts were analysed using Braun & Clark’s 6-step guide for Thematic Analysis [28]: (1) familiarisation with the data occurred during data collection and through rereading transcripts and listening to audio files; (2) initial codes were managed using NVIVO 11. A pragmatic critical realist approach was used. Analysis was data driven and therefore no specific theoretical framework was used; (3) initial codes were combined into broader overarching sub-themes and themes, and a series of schematics were developed to better understand how the themes fitted together; (4) initial themes were refined in collaboration with a second reviewer to ensure that they fit coherently within each theme; (5) themes were further defined and refined, and each theme was assessed for general or systematic agreement/difference by demographic group; (6) a written report was produced. At all stages the first author consulted co-authors to agree coding and interpretation. A study steering group member and patient expert in HIV provided feedback on findings from a patient perspective.

## Results

**Fig. 1:**
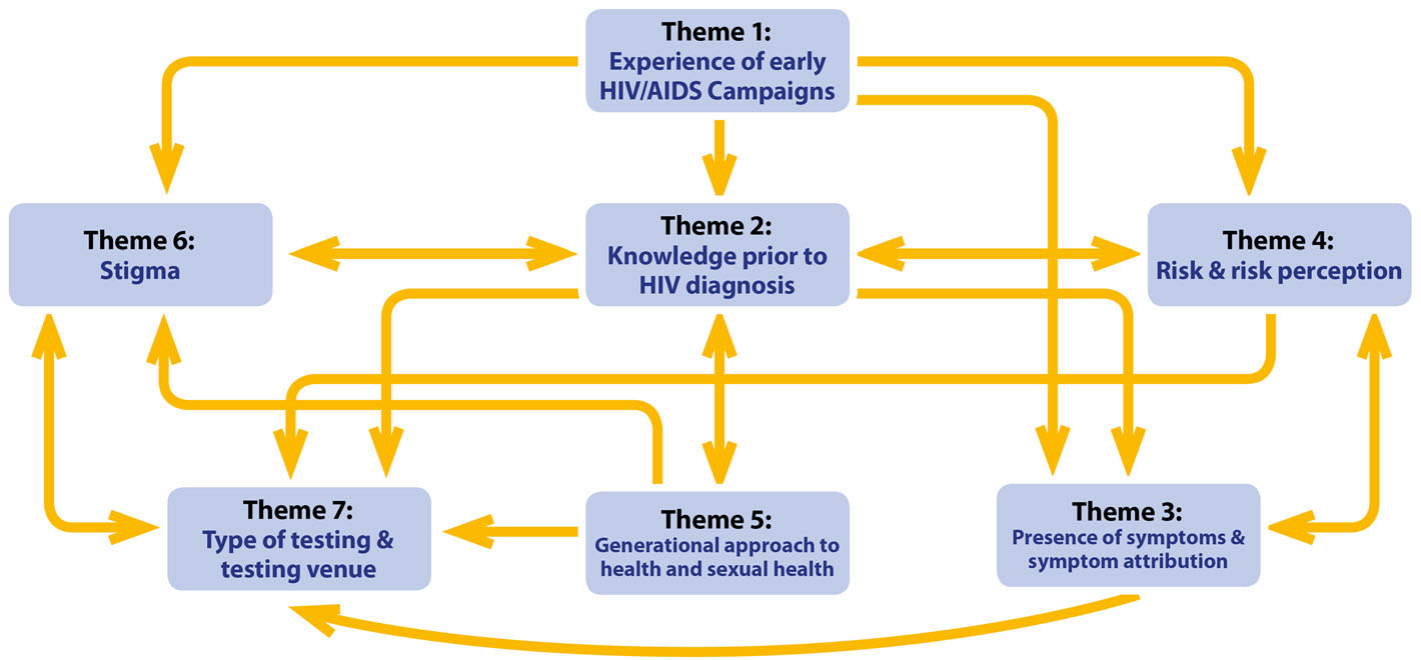
Thematic map of identified themes

Thematic analysis identified 7 major themes associated with testing for HIV in people aged ≥50 years (Fig. 1). Each theme is discussed below and illustrated with quotes from interviewees.

### Theme 1: Experience of the early campaigns

Experience of early HIV/AIDS campaigns underpinned or overlapped with many other themes identified, and was mentioned by almost every participant regardless of demographic group. Participants commonly described how their experience of early HIV/AIDS public health campaigns shaped their beliefs and behaviours. However, the impact these early campaigns had on participants depended on how they identified with the message.

Although heterosexual men and women living in the UK at the time reported being aware of these campaigns, early depictions of HIV as a gay disease left them feeling removed from these messages. Conversely, gay and bisexual men living in the UK and black Africans living in Africa at this time were aware that they belonged to a high risk group as a result of these campaigns. Consequently, these groups discussed how this had reduced their sexual risk taking behaviour or encouraged them to test for HIV. However, this effect diminished once these early campaigns ended:

I was a teenager in the 80s when AIDS first came along. And um, well, publicly anyway. And um, all the horror stories and adverts on TV and on the billboards, and on postage stamps, and, “don’t die of ignorance,” there was all that thing. So, I was brought up with that and I was … I always thought I was really careful. But then as the years go by, maybe you are less careful, you know. [John, 53, White, Gay]

Further, gay and bisexual men, and black African participants commonly described the loss of loved ones at this time, which may be reflective of a higher prevalence of HIV within their communities:

Unfortunately for me in southern Africa, er, it was a proper plague, literally a plague. So you, erm, you had to attend a funeral every week, every weekend, someone who you knew would have passed away. Someone you knew was ill in hospital, so it was like you finished work on a Friday, you know Saturday and Sunday I’m visiting someone in hospital or you were going to bury someone. So it was a real sort of like plague. [Daniel, 55, Black African, Heterosexual]

Universally though, participants discussed their experience of early campaigns in terms of how they shaped their views of HIV. Heterosexual men and women and bisexual men (any ethnicity) commonly reported having no other exposure to HIV-related information since these early campaigns, which tend to be confined to gay venues and media. As a consequence, they held onto preconceptions about HIV until they received their diagnosis. Many felt that a diagnosis of AIDS was a “death sentence” and built a stereotype of who might be at risk of HIV, or what kind of person would be most likely to acquire the infection (Theme 4). For example, many still felt that HIV was a “gay plague” or something which affects people living a certain type of lifestyle; “drug users”, “wrong doers” people without “morals”, “promiscuous” people or people who engaged in “dirty” or “seedy” sex:

I suppose it’s because they still think of it as this is the sort of thing that doesn’t happen to me and it only happens to people who have you know, people who are promiscuous, people who have drugs problems or people who are gay and they just think “It won’t happen to me, I’m just an ordinary person” [Adam, 59, White, Heterosexual]

### Theme 2: HIV Knowledge prior to diagnosis

Poor HIV knowledge prior to diagnosis was mentioned by all participants. They felt that in general people were not well informed about HIV, particularly about advances in treatment. There was agreement that a lack of HIV-related health promotion beyond gay venues and media facilitated this ignorance - particularly among heterosexual men and women:

I was very surprised at how more advanced medical treatment had come since I last thought about HIV. I was amazed that my first reaction was “Oh god I've got my grandchildren, I can't feed them, I can't drink out of the same cup as them or they can't out of me”, and I was very concerned about that [Penny, 75, Heterosexual]

In some cases, participants reported that they did not even know where they could test for HIV. Additionally, several participants felt no need to educate themselves about HIV as they did not think it was something they needed to be aware of (Theme 4):

I never put myself in a category to ever come in contact with HIV, so I’d never educated myself on HIV. I didn’t feel the need to, ‘cause I was never at risk as far as I was concerned [Nicolas, 52, White, Heterosexual]

Conversely, some participants, particularly gay men, felt they did have up to date HIV-related knowledge prior to diagnosis and some reported actively seeking information as a way of keeping themselves up to date. Two heterosexual participants also felt they had up to date knowledge but this was due to work-based training, not because of an active desire to keep up to date.

Knowing where to access HIV-related information or personally knowing people with HIV in the time leading up to their diagnosis was only reported among gay men. Additionally, some gay men felt that people had generally become more aware of HIV in recent years, but this was not mentioned by heterosexual participants:

More recently I mean it’s erm, well people are far more educated about it now. Erm, most people are pretty cool about the whole thing, I don’t think there’s the hysteria, or the hysterics or the neurosis of what people had early on [Chris, 55, White, Gay]

Most participants believed that people living outside high prevalence areas, heterosexual people or older people, who were not traditionally highlighted as risk groups for HIV (Theme 1) had poor HIV knowledge. There was also the belief, particularly among heterosexual participants, that someone only learns about HIV after receiving a positive diagnosis:

No-one knew what HIV was when it was killing people, em, but the first people to get it, I believe, were gay people and that’s what they put it down to. They, you know, and it stuck, and I think it always will, I don’t think it will ever go away. The only time it goes away is if you get it. That’s when you lose that ignorance of it then. [Nicolas, 52, White, Heterosexual]

It was reported that a lack of up to date information, particularly of medical advances, resulted in fear. In some cases - particularly, but not solely among heterosexual participants - it was felt that this fear could lead to test avoidance:

Quite frankly one of the reasons that I spose I didn’t get tested is I was scared to go and get tested [Dennis, 52, White, Bisexual]

Almost every participant felt there was a need for updated HIV-related information to be more widely available. They felt that education campaigns needed to be attention-grabbing, but should be different to the early campaigns which brought fear:

That's how it works with all sorts of things isn't it, until your attention is actually focused on it. You don't really read the information that is coming, actually enough to ... you have to make the information quite ... to grab people's attention quite dramatically in order to get them to take notice. You can't do it passively. [Simon, 61, White, Gay]

### Theme 3: Presence of symptoms and symptom attribution

Whether a participant had experienced physical symptoms in the time leading up to their diagnosis, and what they attributed any symptoms to, influenced decisions to test for HIV.

In the time leading up to their HIV diagnosis, most participants had been unwell, and many had had multiple appointments and/or tests in primary or secondary care. This indicated that there were several missed opportunities for testing. Some participants felt that a lack of continuity of care was a barrier to HIV testing: no one coordinated their care and their doctors treated presenting symptoms rather than taking a full history. This was particularly common in participants who regularly saw more than one clinician:

The lack of consistency means that not one person will sit there and say “Well we’ve tried this, we’ve thought of this”. Nobody did that. It was just: you present, you’re given five minutes, let’s think what the most likely thing is and as I said I was given so much medication ... by various, I had to get, you know, one of the season tickets for prescriptions because they were just prescribing different stuff constantly to me. [Jenny, 57, White, Heterosexual]

Participants in all demographic groups indicated that when their clinician had not mentioned HIV, or had attributed their symptoms to another cause, this presented as a barrier to testing. Because patients trusted the clinician and their knowledge, they did not feel the need to consider other potential causes of disease. However, one participant turned to the internet in order to self-diagnose when she felt her clinician did not take her symptoms seriously:

I also looked it up on the internet and the only thing for weight loss was cancer or AIDS and I, I said to them, you know, “These are the only two things I can have”, and they said that I didn’t have it. [Barbara, 57, White, Heterosexual]

Universally, participants attributed their symptoms to another cause which presented as a barrier to seeking an HIV test. Common symptoms reported by patients were coughing or breathlessness, exhaustion, unexplained weight loss, raised glands, rash, night sweats, vomiting, diarrhoea, herpes sores, swollen joints, loss of appetite, or a general feeling of malaise. In most cases, participants misattributed symptoms to recurrent chest infections or pneumonia, a general cold/influenza, being run down, or cancer. There were several different reasons why participants misattributed their symptoms.. Some felt that although they had engaged in risky behaviour in the past, presenting symptoms were too long after the risk to be HIV-related. Although this was not confined to one demographic group, it was always reported in participants who were not aware that HIV can be asymptomatic or of which symptoms could indicate HIV. Some participants attributed their symptoms to something that was not serious, particularly if they felt well leading up to their diagnosis or prior to symptom onset. Further, when symptoms stopped or when prescribed medications alleviated them, participants did not associate them with HIV. Attributing symptoms to ageing was another sub-theme reported across demographic groups, particularly in patients who reported generalised symptoms such as those associated with a common cold or chest infection, or when these symptoms persisted over prolonged periods:

I just assumed it was a very bad cough. I mean, I think prior to that I'd had a cold and rather foolishly I just thought I was getting older and that's perhaps what happens as you get older, you don't shake these things off in the way that you do when you're younger. [Simon, 61, White, Gay]

Having experience of other health conditions was another barrier to attributing symptoms to HIV. This presented as either having personal experience of a particular health condition and therefore attributing symptoms to this, or having a family history or friend/loved one who had experienced a health condition with similar symptoms, such as cancer or gout

Having had a family of erm, with cancer er, obviously that was probably the first thing in my mind more than HIV then. [Simon, 61, White, Gay]

Some participants suggested that educating people about the symptoms of HIV could increase uptake of HIV testing:

If I’d known that you can have HIV without displaying symptoms I would have still, I would have taken the test instead of like waiting until I had a referral for it. [Oliver, 55, White, Gay]

However, not all participants had experienced symptoms. Some either felt healthy prior to their diagnosis, or did not experience symptoms at all; both were barriers to seeking an HIV test:

Most of the time if people have ... are diagnosed with cancer, it’s because they’ve not been feeling very well. Do you know what I mean, so you’re pushed to go, not because, “Oh I feel fantastic today, I think I’ll go and have a HIV test” [laughing]. You don’t think like that do you? [John, 53, White, Gay]

When participants did experience symptoms which either they or their clinician felt were related to HIV, this led to HIV testing. However, this tended to happen after alternative avenues had already been explored and tests for other potential causes had already been performed.

### Theme 4: Risk and risk perception

Low actual or perceived risk was mentioned by every participant, and resulted in a lack of motivation to seek testing. Heterosexual and bisexual participants additionally felt that they were not the kind of people at risk of HIV:

If you live a certain lifestyle, if you’re an intravenous drug user, or you’re openly gay or whatever maybe you are at higher risk. But I didn’t, I’m neither of those things, so I didn’t think, I didn’t put myself in the category of being at risk I suppose, you know you just don’t do you? [Adam, 59, White, Heterosexual]

Every participant talked about the kind of sex or the type of person who would be at risk of HIV. Commonly this was reported as people who were “gay”, “drug users”, “promiscuous” or people who had “wild” and “unprotected” sex with numerous casual partners, or had risky sex over long periods of time. Not perceiving themselves as fitting the stereotype meant they did not perceive themselves to be at risk of HIV. This seemed to be reinforced by the perception that clinicians also stereotyped them as being low risk, and therefore did not offer HIV testing:

I don’t fit into the demographic, do I? I’m not a young, I’m not a gay person, I’m not African, I’m not any of those things am I? In their mind I’m just a middle aged ... I think they thought I was a middle aged hypochondriac to be honest [Jenny, 57, White, Heterosexual]

There was also universal agreement that older people were at less risk of HIV, either due to a change in lifestyle or due to a perception they are more sensible than younger people. Being in a long term relationship added to the perception that older people were at less risk, a sub-theme more commonly reported among heterosexual participants. However, there was a sense that there had been a change in risk in recent years, mainly due to increasing rates of relationship breakdowns, or the emergence of online dating sites/apps.

Additionally, several participants reported trust within a relationship as important to their perception of risk. Participants who felt their partner was not at risk of HIV because they were “clean” or “nice” did not feel at risk. It was felt that if a partner were positive, then participants trusted them to disclose this or protect them by taking precautions:

I had trust in anyone I was involved with, I trusted them, but in hindsight, I shouldn’t have trusted them, because I didn’t know what they were up to, on their nights out with their friends, I wasn’t involved. [Duncan, 80, White, Bisexual]

Unlike trust which was not confined to one particular demographic group, mistrust was more common among black African participants. Within this group, perceived gendered power differentials within a relationship meant the man would ultimately have control over deciding whether to test. Fear of how a test might harm the relationship meant women did not want to seek a test even if they felt at risk:

That’s also part of our upbringing to a certain extent. The male is a dominant, he does, he’s in charge of whatever is happening, so if you want the relationship you’ve gotta do what he says you know which is unfortunate. I understand that, it’s totally unfortunate, but yeah in our sort of like society you can get away with stupid things like that. Er, “I’m the dominant, this is what we’re gonna do” [Daniel, 55, Black African, Heterosexual]

Denial of risk or apathy were other common sub-themes, although much less common among female participants. This tended to translate either to total denial of risk or weighing up a potential risk of HIV against the benefit of unprotected sex. Closely related to this was a feeling of invincibility, which was most commonly reported in gay and bisexual men. Awareness of belonging to a high risk group (Theme 1) and having lived for so long without contracting HIV left these participants feeling they had a successful prevention strategy. This was reinforced by having HIV tests that had always previously been negative:

There is a certain arrogance that comes from having gone through several decades and not contracting it and saying “Well you've got everything, you know you've got things set up right”. Suddenly you find you haven't. [Simon, 61, White, Gay]

### Theme 5: Generational approach to health and sexual health

A generational approach to health and sexual health was common. This fell into three main sub-themes: how people of their generation approached healthcare and how they prioritised their health; how they felt talking about sexual health/HIV testing; and how the relationship with or personality of the healthcare provider impacted on how they might approach these issues.

#### Approaching healthcare and prioritising health

Some participants, mainly men, felt that older people do not go to the doctor unless they are unwell. However, there was a general view that health screening in older age was common, for example for prostate or breast cancer screening, or for over 50s health checks. As a result they felt more likely to take control of their own health, or be more readily encouraged to test or screen:

My sister and a close friend of me, encouraged me to get, I was about fifty or whatever, encouraged me to go and have you know, a sort of fifty thousand mile service if you like. Go and see the GP, and have a few checks done and I think we’re all encouraged to do that, prostate and other things at that age, and err you should do. So you’re looking after your general health, you know, as you’ve reached obviously well into middle age by then. [Dennis, 52, White, Bisexual]

However, participants tended to worry about things other than HIV, feeling that at their age sexual health was not a priority. They tended to focus on things they knew from targeted health promotion messages or through experience of friends/loved ones. Some participants assumed any blood they had given in either primary or secondary care had been automatically tested for HIV. Further, female participants felt that there was a loss of contact with healthcare in terms of sexual health in older age as the menopause meant they no longer needed to attend for contraception:

The perception for me would be that’s an add-on to those conversations you have around when you… you know go on the pill or… yeah and I haven’t had that sort of conversation with anybody for a very long time. [Jenny, 57, White, Heterosexual]

#### Talking about sexual health/HIV testing

Although some participants felt talking to a clinician about sexual health and HIV testing was potentially embarrassing, there was agreement across all groups that they would not be offended if a HCP brought up this topic of conversation or offered them an HIV test, and most felt they would always agree to a test if it were offered. However, there was a feeling that there was a potential for other people to be offended:

I would never have been upset that somebody had done an HIV test on me with or without my permission. But I know there are some people who have got hang ups about this and they would have felt insulted. So it is not an easy one to get right. [Simon, 61, White, Gay]

Almost all participants felt that there were generational aspects associated with feeling embarrassed to talk about sexual health, which included a lack of sexual education in school, or thinking about sexual health in terms of contraception rather than disease following the sexual revolution. Perceived stigma around gay sex, guilt around sex outside of a long-term relationship or feeling sex was not expected in older age also led to embarrassment:

If you ask my sons who are in their forties, erm, whether they thought their parents still had any sexual activity, they'd be absolutely horrified. Erm, again it is slightly a generational thing. Erm, as you get older people don't expect you to do or to want to do that sort of thing. [Joseph, 68, White, Heterosexual]

Sexual health not being related to the appointment also made it more difficult to talk about sexual health. Conversely, many participants felt that a HCP may feel uncomfortable having a conversation with an older person about sexual health rather than the patient. However, many felt it was the doctor’s duty to ask, and trust in clinicians’ judgement meant participants would generally be happy to test for HIV should the HCP feel it was necessary.

#### Relationship with or personality of the HCP

Despite these barriers, some participants reported facilitators to having a conversation with a HCP around sexual health. This included the HCP initiating the conversation, how the topic is approached by a HCP, or HIV testing being part of other screening. Although not all participants felt a relationship with a HCP was important, the majority either valued a relationship or valued a HCP who was non-judgemental and personable. Although some did value a long-standing relationship, some talked about relationship in terms of “rapport” which might occur even if they did not know the HCP previously:

You can actually go straight to a locum Doctor and feel absolutely put at ease within the first five minutes, it’s obviously one of those things that generates from the Doctor and from the patient, it’s a sort of two-way thing sometimes. [Chis, 55, White, Gay]

Equally it was felt that the personality of the HCP or a bad relationship meant the participant would actively avoid a conversation around sexual health. However a few men reported a long-standing relationship with a HCP might make it more difficult to discuss sexual health, opting instead for a sexual health specialist rather than a general practitioner (GP):

There’s definitely a lot of people that would struggle to do that, even in front of a GP, and possibly more in front of a GP they knew quite well, they’ve been visiting regularly, to start coming up with a topic they never, you know, divulged before, probably extremely hard work [Dennis, 52, White, Bisexual]

### Theme 6: Stigma

Stigma was mentioned by all participants: most commonly in terms of how a person with HIV might be perceived, and stereotypes of high-risk individuals. Participants from all demographic groups felt shame or blame surrounding their diagnosis and as a result feared judgement by friends/family, colleagues or HCPs. The perception that people consider HIV a disease of gay men or ‘dirty’ people was a source of stigma common among heterosexual and bisexual men. The association of gay sex being abnormal or wrong was strongly related to this:

The stigma that’s been put on, “Oh, you’re dirty ‘cause you got it, ‘cause, you know, you’re dirty, gay people”, and it’s, you know, that’s, it’s just the way it came about in the 80s when it started and it’s, it, the stigma stuck and I think it will always be there myself [Nicolas, 52, White, Heterosexual]

Fear of how they would be treated by other people in the light of an HIV diagnosis and resulting social isolation were also described. However, this was more common among female participants, particularly black African women. Female participants also mentioned how stigma might negatively affect current or new relationships.

Additionally, stigma translated into how people felt about using sexual health services. They worried about what the need for sexual health services would say about them and how this would be perceived by others. As a result, many felt uncomfortable using these services, especially when they were clearly labelled as a “sexual health” department, and many feared being seen there by people they knew. However, this was more common among heterosexual participants:

You’re sort of always looking around the room in case someone you know walks in, then they go, “Oh, I saw Nicolas up the Sexual Health Clinic”, you know, then before you know it, rumours go round and that’s what I’m scared of [Nicolas, 52, White, Heterosexual]

For heterosexual participants, the sexual nature of HIV meant it was viewed as socially unacceptable. Gay participants discussed how HIV was different to other sexually transmitted infections (STIs) which were common and easy to catch. An association of HIV as a gay disease or contracted through very high-risk sex resulted in stigma unique to HIV. The treatability of STIs versus the long-term nature of HIV also influenced stigma:

I think because, er, Chlamydia or Gonorrhoea they know that the ... cleared up straight away with tablets and that’s it, they’re done and dusted, they’ve had their, erm, antibiotics so they’re done with that, but I think with HIV it’s because it’s a long term thing and I think that’s what people, that’s what worries people [Oliver, 55, White, Gay]

The majority of participants, regardless of demographic group, perceived there to be an added stigma of being diagnosed with HIV in older age. This was closely related to sex not being expected in older age, particularly high risk sex such as sex with multiple casual partners, which was commonly associated with HIV acquisition. Further, it was felt that older people should know better than to contract HIV, particularly considering they lived through the early era of HIV.

Participants who had experience utilising sexual health services felt that these services were not set up for older people, but instead catered mainly for the needs of younger people:

Making an appointment is easy enough and the staff are always very good but I do sometimes feel, particularly when it is a crowded waiting room and you are with sort of 18 years olds and you are sitting there thinking, well you assume they are thinking “What is this old boy doing here”? [Simon, 61, White, Gay]

### Theme 7: Type of testing and testing venue

The type of HIV testing, such as targeted or routine testing, and the testing venue were both commonly mentioned as important factors associated with testing. Although there was near universal agreement that a test would be accepted if offered, the way it was offered and the setting in which it was offered were important. Participants across demographic groups valued confidentiality regardless of the testing venue. Having a service which was simple, easy or quick were also valued attributes, although was much more commonly reported among gay men.

It was felt, mainly among gay men, that there were benefits of a specialist service and as a result they felt sexual health services were appropriate venues for HIV testing. However, many participants across demographic groups felt general practice would be the most appropriate place for older people to test for HIV. Because participants rarely felt at risk of HIV and therefore did not generally seek a test, the majority felt the most appropriate facilitator to encourage testing would be to add HIV testing into other general screening, making the most of existing clinical contacts. It was felt that this type of screening would become acceptable to patients, normalising HIV testing much in the same way people get used to screening for other conditions such as bowel cancer:

People would get more and more used to it, it’s like anything new isn’t it people, you always get people who say, “Oh I’m not coming back for that, I don’t fancy that”, but you know but generally people would eventually start to come round to it and just go “Yes it’s just another routine check” [Adam, 59, White, Heterosexual]

Participants across demographic groups felt that adding HIV testing into annual health checks or “well man/woman” screening would be an appropriate method to specifically target older people to screen for HIV. Further, it was felt that any blood samples taken should be routinely tested for HIV, although this was more commonly mentioned among gay men.

Offering HIV testing to everyone regardless of their actual or perceived risk was felt to be a facilitator. This was in contrast to targeted screening which some participants felt could cause a patient to feel singled out. Additionally, several participants from across demographic groups felt it was appropriate to test all patients for HIV without prior consent:

I would make it, make it mandatory, that anyone having a blood test for any reason, that HIV is included in that blood test. [Duncan, 80, White, Bisexual]

Most participants felt that home testing or sampling would not be appropriate. Many did not trust the process either in terms of its accuracy, or in how confident they would be in performing the test themselves. Some reported feeling more confident having a face to face interaction with a clinician both to make sure the test was done properly and to be in a safe space should the test come back positive:

I think it’s very dangerous, someone sitting at home, getting an HIV result in front of them, saying “Positive”. [Dennis, 52, White, Bisexual]

Some participants offered suggestions for alternative HIV testing venues. Although gay men acknowledged that outreach in gay venues already existed, some participants suggested offering testing through community pharmacies, through the workplace, or promoting testing through online dating websites.

## Discussion

This analysis identified seven factors associated with testing for HIV in people aged ≥50 years. Although some of these factors have been observed in general populations such as HIV-related knowledge, several such as added stigma in older age, perception of changing HIV risk due to ageing, and the effect of early HIV/AIDS campaigns, are specific to the older age group.

All of the identified factors were interrelated (Fig. 1). In particular, experience of early HIV/AIDS campaigns (Theme 1) directly related to knowledge (Theme 2), symptom attribution (Theme 3,) risk perception (Theme 4), and stigma (Theme 6). It is therefore vital that any educational campaigns targeting older individuals not only include information on medical advances such as the availability of effective therapy, but also dispels any misconceptions people have as a product of these early messages. Almost every theme had a significant impact on the type of testing and testing venue (Theme 7) that older individuals felt comfortable utilising. Therefore, when designing testing programs, it is important to consider that many older individuals do not know where to seek HIV testing, do not feel at risk of HIV, and feel a stigma associated with utilising dedicated sexual health services. Therefore, primary care may be a more appropriate location for older individuals to undergo HIV testing, particularly by combining HIV testing with existing screening (Theme 5). Almost every participant in this study acknowledged a lack of understanding about HIV in the general population. Older individuals had often held on to outdated views formed as a result of the early health promotion campaigns. This lack of up-to-date knowledge was associated not only with stigma, but with a lack of perceived risk whereby participants did not feel the need to seek an HIV test. A recent review of literature indicated that although being offered/encouraged to test for HIV was a facilitator to receiving an HIV test among older adults, preconceptions about older people both in terms of their low risk and how an older person might react to questions regarding risk factors acted as a barrier to test offer from clinicians [27]. Although there is other evidence to support claims that clinicians are less comfortable offering HIV tests to older people [29], in the current study older people were happy to discuss sexual health with their HCP and would generally be happy to accept the offer of an HIV test (Theme 5), even if they did not perceive themselves to be at risk. Furthermore, participants were happy to have an HIV test performed without prior consent when a clinician felt this was necessary, and some believed that any blood taken had automatically been tested for HIV. However although opt-out testing has become increasingly common (particularly in areas of high prevalence), routine testing for HIV without prior consent is not currently recommended [30]. This is in line with testing for other medical conditions, where a HCP is expected to discuss any tests, and obtain consent from a patient prior to any investigation [31]. More research is needed to understand general views about the acceptability of routine opt-out testing. Current UK guidelines recommend that HIV testing should be offered to anyone having a blood test for any reason in an area of high HIV prevalence [32]. Research is needed to better understand whether integrating routine opt-out testing would encourage HCP to perform HIV testing.

The observed mismatch between clinicians’ perceptions and patient expectations regarding the offer of an HIV test may in part be due to stereotyping. This study found that not only did patients form stereotypes of at-risk individuals (which affected their own perception of risk), but they also felt that clinicians stereotyped them to be low risk because of their age. The perception that HIV is a young person’s disease [18], that older people are asexual, or that they find sexual health to be a private topic [33] may all contribute to this. Furthermore, a lack of knowledge about what symptoms may be associated with HIV meant participants often attributed symptoms either to ageing or to a condition of which they already had experience.

A lack of information about HIV beyond gay venues extended to a lack of awareness of where to seek HIV testing. Consequently, many participants felt primary care was an appropriate place for testing. Further, several women reported that ceasing use of hormonal contraception removed a potential opportunity to discuss sexual health with HCPs. Clinicians also feel that appointments for reproductive health allow for a conversation around sexual health that is lost in older age [34]. This highlights a need to address the changing sexual health of older women, and the potential to utilise other clinical interactions, such as consultations for cervical smears or hormone replacement therapy, to discuss sexual health and HIV testing.

Despite commonly held perceptions that older people are less sexually active and at less risk of HIV, sexuality is now considered a factor contributing to successful ageing, and sexual activity is positively associated with overall health [33]. Relationship transitions are increasingly common in older age [35], this in addition to lower condom use in older age [36], and physiological changes such as vaginal dryness [37], may actually put older people at increased risk of HIV.

Participants reported that they had a degree of trust in their partner, either that they were HIV negative, or that a positive partner would inform them or take steps to protect them. An association between trust and a lack of condom use has been reported in past research, albeit among younger people [38, 39].

Stigma was another significant barrier to testing. Although HIV-related stigma can been observed in the general population [40], participants reported an added stigma associated with being diagnosed with HIV in older age. This suggests that services may need to adapt to better serve older people. Having dedicated older person’s sessions at sexual health clinics might be one way to encourage older people to use current services. Stigma related to sexual orientation or asexuality in older age were both identified in the current study. The routine offer of HIV testing, despite actual or perceived risk, could encourage testing in older individuals who may not feel comfortable to disclose sexual risk behaviours or sexual orientation, and may also circumvent HCPs embarrassment discussing sexuality [23, 24]. Another suggestion to encourage testing in this group might be to utilise HIV self-sampling. Although this has been shown to be particularly acceptable to people who do not want to use sexual health services or have never previously tested for HIV [41, 42], participants in this study did not consider it acceptable (Theme 7). One of the main barriers was concern about test accuracy or that they would perform the test incorrectly. Despite this, previous research in younger populations has found that self-testing is easy to perform and to read [42].

### Strengths and limitations

A key strength of the current study is that it addressed a lack of knowledge of factors associated with testing for HIV in people aged ≥50 by including people aged up to 80 years. Although this study included participants from a range of demographic groups (in terms of ethnicity and sexual orientation) and in areas of high and low HIV prevalence, all participants were recruited from South East England and therefore their views might not be representative of individuals in other parts of the country. Further, participants took part in the study between 1 and 36 months from receiving an HIV diagnosis. It is therefore acknowledged that views and experiences expressed may differ depending on the length of time since receiving an HIV diagnosis.

### Clinical implications and recommendations

Identifying unique factors associated with a decision to test for HIV in older age has the potential to translate into interventions to increase testing within this group. However, to increase earlier testing, interventions should focus on tackling these barriers and designing services which are flexible to meet the different needs of unique demographic groups. These include increasing knowledge; addressing stigma; assessing different testing methods; and adapting sexual healthcare delivery to better meet the needs of older people.

The findings suggest that older people need to be better informed about HIV in terms of potential risk, medical advances and how to access testing services. Increased awareness may also help to reduce stigma and help in the recognition of symptoms, both of which may increase testing overall and testing at an earlier disease stage. However, any educational campaigns need to be active and ongoing since a lack of perceived risk was also found to be associated with a lack of HIV-related information seeking. However, it was acknowledged that early HIV/AIDS campaigns promoted fear and stigma, and as a result any new messages would have to be significantly different. Further, chosen modality of communication would have to be suitably accessible to the older population.

National HIV testing week is successful at promoting HIV testing [43]. However, it is targeted at younger groups and MSM and as a result, older people in this study felt it was not relevant to them. This campaign may be a good opportunity to target the older group by tailoring promotion materials, for example to contain pictures of older people, or displaying materials at venues more appropriate to this group.

This study indicated that current services may not be appropriate to meet the sexual health needs of older people. There was general agreement that incorporating HIV testing into current health screening would be an appropriate way to encourage testing, particularly since older adults in this study indicated they would accept an HIV test if offered by a HCP. This is a finding consistent with other research indicating that a major factor associated with HIV testing among older populations is the offer of a test by a HCP [27]. This might include offering HIV tests in primary care during well man or well woman clinics, or during appointments for other screening, such as blood pressure monitoring. The ‘making every contact count’ initiative offers some insight into how using existing clinical contacts to disseminate information to service users might be an appropriate way of reaching this population [44]. In addition, this study found that testing for HIV without prior explicit consent was appropriate to many individuals, and so addressing current consenting procedures may be an important step into how routine testing occurs currently in inpatient and outpatient settings. However, any potential interventions will need to include input from service users as well as service providers to better identify gaps in service provision and to make sure services are appropriate.

## Conclusion

This study identified unique barriers associated with HIV testing within the older population. People aged ≥50 years often do not perceive themselves to be at risk of HIV, associated with outdated beliefs. Further, stigma and a lack of knowledge of how to access HIV testing suggest a need for health promotion and suggest current sexual health services may need to adapt to better meet their needs.

## Abbreviations

GP: General practitioner
HCP: Healthcare provider
MSM: Men who have sex with men
STI: Sexually transmitted infection
UNAIDS: the Joint United Nations Programme on HIV/AIDS
